# Chemical Profiling and Biological Properties of Extracts from Different Parts of *Colchicum Szovitsii* Subsp. *Szovitsii*

**DOI:** 10.3390/antiox8120632

**Published:** 2019-12-11

**Authors:** Gabriele Rocchetti, Biancamaria Senizza, Gokhan Zengin, Murat Ali Okur, Domenico Montesano, Evren Yildiztugay, Devina Lobine, Mohamad Fawzi Mahomoodally, Luigi Lucini

**Affiliations:** 1 Department for Sustainable Food Process, Università Cattolica del Sacro Cuore, Via Emilia Parmense 84, 29122 Piacenza, Italy; gabriele.rocchetti@unicatt.it (G.R.); biancamaria.senizza@unicatt.it (B.S.); luigi.lucini@unicatt.it (L.L.); 2 Department of Biology, Science Faculty, Selcuk University, Campus, 42130 Konya, Turkey; murataliokur@hotmail.com; 3 Department of Pharmaceutical Sciences, Food Science and Nutrition Section, University of Perugia, Via S. Costanzo 1, 06126 Perugia, Italy; 4 Department of Biotecnology, Science Faculty, Selcuk University, Campus, 42130 Konya, Turkey; 5 Department of Health Sciences, Faculty of Science, University of Mauritius, 230 Réduit, Mauritius; devina.lobine@gmail.com (D.L.); f.mahomoodally@uom.ac.mu (M.F.M.)

**Keywords:** *Colchicum*, polyphenols, alkaloids, metabolomics, antioxidants, enzyme inhibition

## Abstract

Like other members of the *Colchicum* genus, *C*. *szovitsii* subsp. *szovitsii* is also of medicinal importance in Turkish traditional medicine. However, its biological properties have not been fully investigated. Herein, we focused on the evaluation of the in vitro antioxidant and enzyme inhibitory effects of flower, root and leaf extracts, obtained using different extraction methods. In addition, a comprehensive (poly)-phenolic and alkaloid profiling of the different extracts was undertaken. In this regard, ultra-high-performance liquid chromatography coupled with quadrupole time-of-flight mass spectrometry (UHPLC-QTOF-MS) allowed us to putatively annotate 195 polyphenols and 87 alkaloids. The most abundant polyphenols were flavonoids (83 compounds), whilst colchicine and 2-demethylcolchicine were some of the most widespread alkaloids in each extract analyzed. However, our findings showed that *C. szovitsii* leaf extracts were a superior source of both total polyphenols and total alkaloids (being, on average 24.00 and 2.50 mg/g, respectively). Overall, methanolic leaf extracts showed the highest (*p* < 0.05) ferric reducing antioxidant power (FRAP) reducing power (on average 109.52 mgTE/g) and 2,2-diphenyl-1-picrylhydrazyl (DPPH) radical scavenging (on average 90.98 mgTE/g). Interestingly, each *C*. *szovitsii* methanolic extract was more active than the water extracts when considering enzymatic inhibition such as against tyrosinase, glucosidase, and acetylcholinesterase (AChE). Strong correlations (*p* < 0.01) were also observed between polyphenols/alkaloids and the biological activities determined. Multivariate statistics based on supervised orthogonal projections to latent structures discriminant analysis (OPLS-DA) allowed for the detection of those compounds most affected by the different extraction methods. Therefore, this is the first detailed evidence showing that *C. szovitsii* subsp. *szovitsii* might provide beneficial effects against oxidative stress and the associated chronic diseases. Nevertheless, the detailed mechanisms of action need to be further investigated.

## 1. Introduction

Medicinal plants have historically proven their value as sources of molecules with therapeutic potential, and even in this new era of highly allopathic medicine, herbal medicines are an area of focus for researchers around the world to complement modern drugs and as sources of novel drug leads [1]. The estimated market of pharma products in 2022 amounts to $1.12 trillion, indicating the rising demand for pharmaceutical products. To meet the global healthcare needs, the World Health Organization (WHO) recommends the practice of the traditional system as it is affordable and culturally acceptable. Furthermore, the adverse effects and toxicity of modern drugs have revived the use of alternative systems of medicines, which has led to the drastic development of the herbal drug industry [1,2,3]. In recent years, plants have been gaining much attention among researchers to discover new drugs or modify existing drugs, in order to manage the increasing incidence of chronic disease. Currently, up to 50% of overall pharmaceutical drugs are derived from plants [3].

One of the important medicinal plants are the Colchicum species, which have been traditionally used more than 3000 years ago to treat various ailments by different ethnic groups. Colchicum is a valuable genus (family: Colchicaceae) with approximately 100 species, mainly distributed in Mediterranean countries, South Africa, and the Caucasus [4]. The name Colchicum is derived from ‘Colchi’, an ancient region on the eastern shore of the Black Sea, and indicates its origin [5]. Colchicum species bear stemless and crocus like flowers, which mainly bloom in autumn, and hence some species are commonly known as autumn crocus, meadow saffron, or naked lady [4,5].

The medicinal value of the Colchicum species is ascribed to the presence of tropolonic alkaloids, colchicinoids, mainly colchicine [6]. Ancient Greek physicians used the plant as a therapeutic agent for gout. In India and Africa, various preparations of colchicum are still used traditionally to treat gastroenterological, musculoskeletal, and cutaneous diseases. Colchicine and its natural analogues are used clinically for the treatment of several disorders such as Familial Mediterranean Fever, amyloidosis, Behcet’s disease, cirrhosis, psoriasis, and many other dermatological diseases [4,7]. However, the low therapeutic index of this alkaloid limits its use in therapy. Demecolcine, another major alkaloid, has lower toxicity than colchicine and has been successfully used in the treatment of myeloid leukemia and Hodgkin’s syndrome. Pharmacological studies have showed that the Colchicum species possess antioxidant [8,9,10], antibacterial [8,9], acetylcholinesterase [11], anti-inflammatory [12], and antiarthritic [13] properties.

Turkey is one of the richest regions for the number of Colchicum species. The genus is represented by 45 species in Turkey, which makes the country a major center for the Colchicum species, not only because of numbers, but also because of the high rates of endemism (35%) [14]. In the continuation of our investigations into plants with therapeutic properties, we have now studied the pharmacological properties of *Colchicum szovitsii* subsp. *szovitsii*, a spring flowering species from Turkey [4]. To the best of our knowledge, previous phytochemical studies have focused on the alkaloid content of *C. szovitsii* [15,16,17]. Sevim ve ark. [11] investigated the anti-cholinesterase and antioxidant activities of methanolic extracts from bulbs and seeds of *C. szovitsii* subsp. *szovitsii* and observed that only the seed extracts were potent against butyrylcholinesterase (BChE) and have displayed antioxidant activity by means of the 2,2-diphenyl-1-picrylhydrazyl (DPPH) assay. Therefore, this is a first detailed investigation of the flowers, roots, and leaves of *C. szovitsii* for their pharmacological attributes and chemical content. Biological investigations comprised antioxidant and enzyme inhibitory (cholinesterases, tyrosinase, α-amylase, and α-glucosidase,) assessment, and ultra-high-performance liquid chromatography mass spectrometry (UHPLC-MS) analysis was performed in order to provide detailed insights into the polyphenol and alkaloid profiles of extracts from different parts (flowers, roots, and leaves) of *C. szovitsii* subsp. *szovitsii.*

## 2. Materials and Methods

### 2.1. Plant Material and Preparation of Extracts

The plant materials of *C. szovitsii* subsp. *Szovitsii*, namely the flowers, leaves, and roots, were collected at Konya in Turkey in 2019 (The plateau of Bartlı, Bozkır village, Konya, GPS: 37°10’.948’’N, 32°05’.274’’E, 1340 m; collection date: 23.02.2019). The plant materials were collected and identified by botanist Dr. Evren Yildiztugay (Selcuk University, Department of Biotechnology, Konya, Turkey, voucher number: EY-2970). The plant parts were allowed to dry in a well-ventilated area away from direct sunlight (about 10 days).

To obtain extracts, we performed infusion, maceration, and Soxhlet techniques. In the infusion technique, the plant materials (5 g) were kept with 100 mL of boiled water for 20 min and then filtered. In the maceration technique, the plant materials (5 g) were mixed with 100 mL of both methanol and water for 24 h at room temperature. In the Soxhlet technique, the plant materials (5 g) were extracted with 100 mL methanol by using Soxhlet apparatus for 6 h. Extracts were obtained by using a vacuum evaporator. Water extracts were lyophilized, and all extracts stored in a refrigerator [18,19].

### 2.2. Chemicals

The chemicals were purchased from Sigma-Aldrich (Darmstadt, Germany). These were: 2,2′-azino-bis(3-ethylbenzothiazoline-6-sulfonic acid (ABTS), 1,1-diphenyl-2-picrylhydrazyl (DPPH), gallic acid, rutin, caffeic acid, electric eel AChE (type-VI-S, EC 3.1.1.7), horse serum BuChE (EC 3.1.1.8), galantamine, acetylthiocholine iodide (ATChI), butyrylthiocholine chloride (BTChI) 5,5-dithio-bis(2-nitrobenzoic) acid (DTNB), tyrosinase (EC1.14.18.1, mushroom), glucosidase (3.2.1.20, from *Saccharomyces cerevisiae*), amylase (3.2.1.1, from porcine pancreas), sodium molybdate, sodium nitrate, sodium carbonate, Folin–Ciocalteu reagent, HCl, NaOH, trolox, EDTA, neocuproine, cupric chloride, ammonium acetate, ferric chloride, 2,4,6-Tris(2-pyridyl)-s-triazine (TPTZ), ammonium molybdate, ferrozine, ferrous sulphate hexahydrate, kojic acid, and acarbose. 

### 2.3. Spectrophotometric Analyses

The total bioactive compounds were determined colorimetrically as described previously [20,21]. For total phenolic content (TPC), a sample solution (50 µL) was mixed with 100 mL of Folin–Ciocalteu reagent (1:9, v/v) and the mixture was allowed to stand for 3 min at room temperature before sodium carbonate (75 µL, 2%) was added. The mixture was then incubated for 2 h at room temperature and the absorbances were recorded at 765 nm in a microplate reader. The results were expressed as the standard compound (gallic acid (mg GAE/g dried extract)).

For total flavonoid content (TFC), a sample solution (200 µL) was mixed with 100 mL of AlCl_3_ (%2, methanolic) and the mixture was kept for 15 min at the room temperature. The absorbances were then recorded at 415 nm in a microplate reader. The results were expressed as the standard compound (rutin (mg RE/g dried extract).

For total phenolic acid content (TPAC), a sample solution (50 µL) was mixed with Arnow reagent (sodium molybdate (10%) and sodium nitrite (10%)). Acid solution (HCl, 0.5 M) was then added to this mixture and incubated for 5 min at the room temperature. 100 µL of NaOH (8.5%) was then added to the mixture and was further incubated for 10 min at the room temperature. The absorbances were recorded at 490 nm in a microplate reader. The results were expressed as the standard compound (caffeic acid (mg CE/g dried extract).

### 2.4. Untargeted Profiling of Polyphenols and Alkaloids by Untargeted Metabolomics

The untargeted phenolic profiling of the different *C. szovitsii* extracts was investigated by means of ultra-high-pressure liquid chromatography (Agilent 1290 HPLC liquid chromatograph; Agilent Technologies, Santa Clara, CA, USA) coupled to quadrupole-time-of-flight mass spectrometer (Agilent 6550 iFunnel; Agilent Technologies, Santa Clara, CA, USA). The experimental conditions for the analysis of plant extracts by means of untargeted metabolomics were optimized as described in previous works [22,23,24]. The mass spectrometer acquired ions in the range 100–1200 m/z in positive scan mode. An in-house database built combining Phenol-Explorer 3.6 with some of the most important alkaloids reported in literature on Colchicaceae was then used for annotation purposes, exploiting the entire isotopic profile (i.e., combining monoisotopic accurate mass, isotopic ratios, and spacing). Therefore, the approach used was based on a Level 2 of identification (i.e., putatively annotated compounds), as set out by the COSMOS Metabolomics Standards Initiative. Furthermore, Agilent Profinder B.06 software was used for post-acquisition data filtering, retaining only those compounds identified within 100% of replications in at least one condition. Thereafter, to provide more quantitative information, the polyphenols were first ascribed into classes and subclasses, and then quantified using standard solutions (80/20, v/v methanol/water) of pure standard phenolic compounds analyzed with the same method [25]. The following phenolic classes were targeted: anthocyanins (quantified as cyanidin equivalents), flavones (quantified as luteolin equivalents), flavonols and flavanols (quantified as catechin equivalents), lignans (quantified as sesamin equivalents), alkylphenols (quantified as 5-pentadecylresorcinol equivalents), stilbenes (quantified as resveratrol equivalents), low-molecular-weight phenolics (quantified as tyrosol equivalents), and phenolic acids (quantified as ferulic acid equivalents). Finally, a calibration curve of sanguinarine (Sigma grade, Sigma-Aldrich, S. Louis, MO, USA) was used to estimate the total alkaloid content. The results were finally expressed as mg equivalents/g dry matter. 

### 2.5. Determination of Antioxidant and Enzyme Inhibitory Effects

For antioxidant capacity, different test systems including radical quenching, reducing power, phosphomolybdenum, and ferrous ion chelating were employed. The details of the methods are described in our earlier paper [20]. The result was reported as trolox equivalents (mg TE/g extract) and ethylenediamine tetraacetic acid (EDTA) equivalents (for ferrous ion chelating; mg EDTAE/g extract). For enzyme inhibitory effects, key enzymes for global health problems were selected, namely α -amylase and α-glucosidase, acetylcholinesterase (AChE), butyrylcholinesterase (BChE) and tyrosinase and the inhibitory activities were compared to standard drugs (acarbose for amylase and glucosidase; galatamine for AChE and BChE; kojic acid for tyrosinase) [20]. 

### 2.6. Statistical Analysis 

A one-way analysis of the variance (ANOVA) was performed considering data from each assay and using the software PASW Statistics 26.0 (SPSS Inc., Chicago, IL. USA), followed by a Duncan’s post hoc test (*p* > 0.05). Pearson’s correlations (*p* < 0.05; two-tailed) were also calculated using PASW Statistics 26.0. Afterward, the metabolomics-based dataset exported from Mass Profiler Profession B.12.06 (Agilent Technologies, Santa Clara, CA, USA) was elaborated into a second software, namely SIMCA 13 (Umetrics, Malmo, Sweden) for supervised orthogonal projections to latent structures discriminant analysis (OPLS-DA), according to previously reported works [24]. The variables selection method VIP (i.e., variables’ importance in projection) was used in order to evaluate the importance of each phenolic compound in discriminating the different extraction techniques, and to select those having the highest discrimination potential (VIP score >1.2).

## 3. Results and Discussion

### 3.1. Phytochemical Composition and Discrimination of the Different Extraction Methods

Natural phenolic compounds are extensively occurring plant secondary metabolites that consist of an aromatic ring bearing at least one hydroxyl group. Up until now, more than 8000 phenolic compounds from plants have been reported. These phytochemicals are recognized for the array of pharmacological attributes they possess such as antioxidant, antibacterial, antiviral, anti-allergenic, cardioprotective, neuroprotective, and anticancer activities [26,27]. In this direction, the phenolic content is presented as an important indicator of biological attributes of plant extracts. In recent years, international research has focused on the importance of extraction methods for the recovery of polyphenolic compounds [28,29]. 

In the present study, the polyphenolic composition of different extracts of *Colchicum szovitsii* subsp. *szovitsii* flowers, roots, and leaves were determined in terms of total phenolic content (TPC), total flavonoid content (TFC), and total phenolic acid (TPAC), as presented in Supplementary Table S1. For the flower extracts, the aqueous macerated extract (40.70 mgGAE/g) was found to have the highest TPC, while the methanolic extract (64.88 mgRE/g) obtained using the Soxhlet method, followed by the methanolic macerated extract (63.03 mgRE/g) displayed the highest TFC. A modest TPAC was observed for all the studied flower extracts. With regard to root extracts, the methanolic extract obtained using the Soxhlet method (13.67 mgGAE/g) displayed the highest TPC, followed by the aqueous macerated extract (11.83 mgGAE/g). Low TPC and TPAC were observed for all of the studied extracts. The leaf extracts showed notable TPC, with a value ranging from 36.55 and 17.50 mgGAE/g. Noteworthy TFC and TPAC were observed for leaf methanolic extracts obtained using the maceration (TFC: 173.65 mgGAE/g; TPAC: 13.21 mgCE/g) and Soxhlet (TPC: 169.04 mgGAE/g; TPAC: 12.38 mgCE/g) techniques. Overall, when considering leaves, the methanolic extracts obtained using the Soxhlet and maceration methods were found to possess the highest phenolic contents. 

Thereafter, untargeted metabolomics based on UHPLC-QTOF mass spectrometry was used to depict the phytochemical composition (i.e., polyphenols and alkaloids) of the different *Colchicum szovitsii* extracts. Based on the experimental data, 282 compounds were putatively annotated according to a Level 2 of identification (i.e., putatively annotated compounds), with 87 compounds being alkaloids and the remainder being anthocyanins, flavanols, flavones, flavonols, phenolic acids, lignans, stilbenes, and other compounds (including alkylphenols and lower-molecular-weight compounds). A comprehensive list containing each compound annotated together with its composite mass spectrum is provided in the Supplementary Materials. Overall, the *Colchicum szovitsii* flower extracts were very abundant in colchiceine, 2-demethylcolchicine, androbine, and gloriosine, followed by glycosidic forms of pelargonidin and cyanidin (anthocyanins), chrysoeriol 7-o-apiosyl-glucoside and luteolin (flavones), galangin and quercetin 3-o-rhamnosyl-galactoside (flavonols), and dimethylmatairesinol (lignan). By looking to the most abundant compounds characterizing *Colchicum szovitsii* root extracts, it is important to mention 10 alkaloids, namely α-, β-, and γ-lumicolchicine, colchicine, allocolchicine, N-formyldemecolcine, 2-demethylcolchicine, gloriosine, androbine, and cornigerine (Supplementary Materials). With respect to the polyphenol group, the root extracts were rich in some lignans (i.e., arctigenin, secoisolariciresinol, anhydro-secoisolariciresinol and dimethylmatairesinol), followed by some low-molecular-weight phenolics (i.e., rosmanol and rosmadial). Regarding *Colchicum szovitsii* leaf extracts, we mainly detected alkaloids such as colchiceine, 2-demethylcolchicine, androbine, and cornigerine (supplementary material), followed by delphinidin 3-o-glucosyl-glucoside and cyanidin 3-o-(6’’-caffeoyl-glucoside) (anthocyanins), hesperetin (flavones), quercetin 4’-o-glucoside (flavonols), dimethylmatairesinol (lignans), and rosmanol (phenolic terpenes). Overall, our findings are difficult to compare with the existing literature; in fact, no similar works focusing on Colchicaceae are based on comprehensive untargeted metabolomics focused on polyphenols and alkaloids. However, according to the reviewed literature [4], the main phenolic compounds characterizing the *Colchicum* species in Turkey are mainly phenolic acids (both hydroxybenzoic and hydroxycinnamics), followed by flavones (mainly luteolin equivalents). This was particularly true when considering the flower, leaf, seed, and corm extracts of the following species: *C. baytopiorum*, *C. bornmuelleri, C. macrophyllum*, *C. speciosum*, *C. triphyllum*, and *C. turcicum*. 

Furthermore, a semi-quantitative approach based on representative phenolic and alkaloid standard compounds was used, and the results are reported in Table 1. With regard to polyphenols, flavonols, phenolic acids, and tyrosol equivalents displayed higher contents (*p* < 0.05) when compared to the remaining phenolic classes, independently from the investigated *C. szovitsii* parts. Overall, maceration based on water and methanol was found to promote the highest cumulative phenolic recovery in flower extracts, recording 17.53 and 16.17 mg/g, respectively. Different findings were obtained for the root extracts (Table 1); in fact, the highest phenolic contents were obtained when methanol was used as the extraction solvent, being 10.79 mg/g for Soxhlet and 10.14 for maceration. Regarding leaves, similar cumulative phenolic contents were detected for samples extracted by the infusion, methanolic maceration, and Soxhlet methods (on average 30.63 mg/g). Regarding total alkaloids (quantified as mg sanguinarine equivalents/g dry matter), higher contents were recorded in the *C. szovitsii* leaf extracts, with an average of 2.50 mg/g (Table 1). In particular, the lowest (*p* < 0.05) total alkaloid content was recorded in flower extracts obtained by water maceration, whilst using methanol as the extraction solvent was found to promote a better recovery of these compounds in root extracts, on average 1.82 mg/g. Therefore, the semi-quantitative findings by UHPLC-QTOF mass spectrometry suggest that *C. szovitsii* leaf extracts are the best source of both polyphenols and alkaloids. 

**Figure 1:**
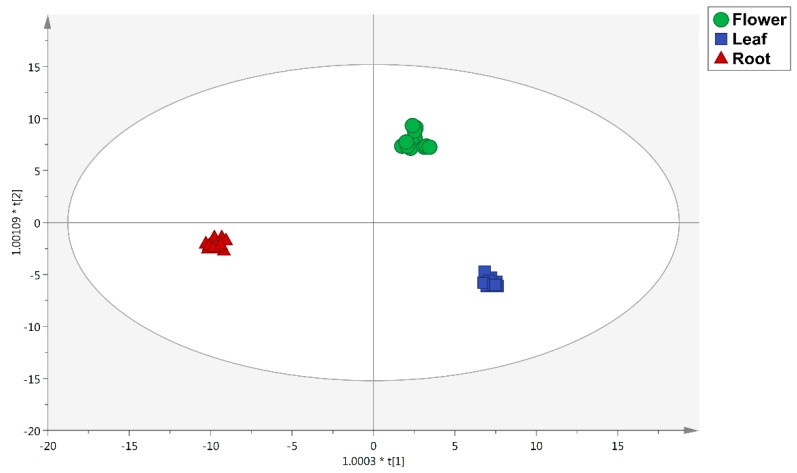
Orthogonal projections to latent structures discriminant analysis (OPLS-DA) score plot built according to polyphenol and alkaloid profiling and considering the different parts of *C. szovitsii* as class membership criteria.

In addition to the untargeted profiling of polyphenols and alkaloids, a supervised multivariate statistical approach was used to depict the compounds mainly related to discrimination purposes, when considering both plant material and the extraction type. In particular, an orthogonal projection to latent structures discriminant analysis (OPLS-DA) was carried out, and then the most discriminant compounds allowing sample separation were selected by means of the “variables importance in projection” (VIP) method. The first OPLS-DA model carried out considering the three different *Colchicum szovitsii* parts is reported as Figure 1. As can be observed, a very good degree of separation according to both polyphenol and alkaloid composition was obtained. In particular, the first latent vector [1] discriminated the flower from the leaf and root extracts, whilst the second latent vector t [2] allowed the separation between root from leaf and flower extracts (Figure 1). The OPLS-DA carried out on the different *Colchicum szovitsii* plant materials was characterized by more than acceptable model parameters, being goodness-of-fit (R^2^Y) = 0.99 and goodness-of-prediction (Q^2^Y) = 0.99. Afterward, the variables importance in projection (VIP) selection method was combined with fold-change analysis in order to monitor the significative differences in the abundance of each discriminant marker detected. The results are reported in the Supplementary Materials. Overall, 61 compounds were characterized by a VIP score >1, with a clear abundance of isomeric forms of alkaloids (34 compounds) and flavonoids (16 compounds). Interestingly, looking to the LogFC values, some compounds were found to possess a higher discriminant potential, according to the plant material analyzed. In this regard, *Colchicum szovitsii* root extracts were characterized mainly by the following markers: specioseine (VIP score = 1.21), 8-prenylnaringenin (VIP score = 1.36), 6’’-o-acetylglycitin (VIP score = 1.28), conidendrin (1.36), Todolactol A (VIP score = 1.35), 3,4-DHPEA-EDA (VIP score = 1.28), and caffeic acid (VIP score = 1.25). Regarding the leaf extracts, several isomeric forms of alkaloids were characterized by high discrimination potentials, with the maximum LogFC values recorded for androcimine/szovitsamine and colchiritchine. Finally, the *C. szovitsii* flower extracts showed a smaller number of discriminant markers, recording the highest variations for the alkaloids colchicoside (VIP score = 1.31) and speciosine (VIP score = 1.22), followed by the isoflavone 6’’-o-acetyldaidzin (VIP score = 1.23).

**Figure 2:**
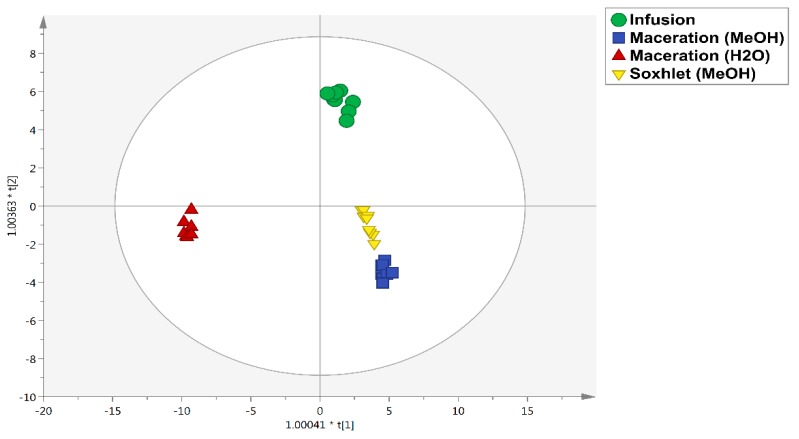
Orthogonal projections to latent structures discriminant analysis (OPLS-DA) score plot built according to polyphenol and alkaloid profiling and considering the different extraction methods as class membership criteria.

The second OPLS-DA model was then carried out in order to outline the effect of the different extraction methods on the chemical profile of the *C. szovitsii* extracts. The score plot obtained considering the extraction method as class membership criterium is reported in Figure 2. As can be seen from the figure, a clear effect of the extraction solvent is depicted, with the methanolic maceration and Soxhlet methods clustered together, whilst the infusion and water maceration extractions were characterized by the most specific phenolic and alkaloid profiles. The variables selection method VIP was used again to list the most discriminant metabolites (i.e., those compounds most affected by the different extraction conditions). In this regard, a comprehensive list reporting the VIP compounds and their corresponding LogFC values is reported in the Supplementary Materials. Overall, 20 compounds were found to possess the highest discrimination potential, with an abundance of flavonoids (eight compounds), followed by phenolic acids (five compounds), and low-molecular-weight phenolics (four compounds). Interestingly, we found that maceration in methanol was able to promote the greatest recovery of the alkaloid jolantine (VIP score = 1.55), while macerated water extracts allowed the best recovery of the polyphenols xanthohumol/isoxanthohumol (VIP score = 1.23), 5-tricosenylresorcinol (VIP score = 1.27), and feruloyl tartaric acid. In fact, jolantine possessed a LogFC of 21.09 (for methanolic macerated extracts) and 20.83 (for methanolic Soxhlet extracts) when compared with infusion extracts (Supplementary Materials). Similar results in terms of the recovery of bioactives were obtained by comparing the methanolic and water macerated extracts (Table S1). Therefore, as a general consideration, the fold-change analysis revealed that flavonoids were the *Colchicum* compounds most affected by the extraction solvents. However, as also reported in our previous work [25,28], the extraction solvent should be carefully selected in order to promote a selective extraction of bioactive compounds.

### 3.2. In Vitro Antioxidant Activity

Reactive oxygen species (ROS), produced at low levels during aerobic metabolism, are essential mediators of important cellular functions and the endogenous antioxidant defense systems of the body have the capacity to prevent any harmful effects. However, at high concentration, ROS results in oxidative damage of macro-molecules, which is extremely harmful to the organism. Studies have demonstrated the implication of ROS in the development and progression of several life threatening diseases including diabetes, neurodegenerative, cancer, atherosclerosis, and cardiovascular diseases [30,31]. Antioxidants are molecules that avert the harmful effects of ROS by inhibiting the oxidation of oxidizable substrates. Naturally occurring antioxidant molecules have received significant attention in both usages and research studies; these have been employed in medical and pharmaceutical products as substitute compounds for synthetic antioxidants, which have been proven to pose adverse effects [27].

In the current study, we investigated the antioxidant capacities of *C. szovitsii* subsp. *szovitsii* flowers, roots, and leaves extracts by assaying their total in vitro antioxidant capacity (phosphomolybdenum assay), DPPH and [2,2’-azinobis-(3-ethylbenzothiazoline-6-sulfonate)] (ABTS) scavenging capacity, ferric and cupric reduction activity, and metal chelating activity (Table 2). Based on the experimental findings, it was observed that all of the studied extracts had a modest total antioxidant capacity. Radical scavengers can prevent free radical induced macromolecules or tissue damage by directly neutralizing free radicals and accepting or donating electron(s) to eliminate the unpaired condition of the radical [32]. Among the different flower extracts of *C. szovitsii* subsp. *szovitsii* tested in this study, the infused (53.20 mgTE/g) and aqueous macerated (52.76 mgTE/g) extracts were found to be the best DPPH scavengers. For the root extracts, the methanolic extracts obtained using the Soxhlet and maceration methods had the highest free scavenging potentials with the DPPH assay (Table 2). Similarly, among the leaf extracts of *C. szovitsii* subsp. *szovitsii*, remarkable free scavenging ability was noted with the methanolic extracts obtained using Soxhlet and maceration (maceration: 91.59 mgTE/g; Soxhlet: 90.37 mgTE/g). For the ABTS assay, a similar trend was observed for flowers and roots, whilst for the leaf extracts, the highest DDPH quenching ability was observed with the infused extract (66.63 mgTE/g).

Reducing power is considered as one of the most important antioxidant mechanisms and this corresponded to the electron-donating capacity of antioxidant ability [32]. In the current study, we found that among the root extracts, the aqueous macerated extract displayed the highest cupric and ferric reducing power, with values of 105.87 and 95.22 mgTE/g, respectively. For the roots, the methanolic extract was potent for the cupric reducing antioxidant capacity (CUPRAC) assay, while the aqueous macerated extract showed the best activity for the FRAP assay. Among all the leaf extracts, the methanolic extracts obtained using the Soxhlet (CUPRAC: 131.03 mgTE/g; FRAP: 132.14 mgTE/g) and maceration (CUPRAC: 110.82 mgTE/g; FRAP: 108.23 mgTE/g) methods of *C. szovitsii* subsp. *szovitsii* were the most potent reducing agents.

Furthermore, we also assessed the metal chelating effect of the different extracts of *C. szovitsii* subsp. *szovitsii*. Transition metal ions such as Fe^2+^ accelerate ROS production in the body. Therefore, the Fe chelating activity of a compound may be related to its antioxidant capacity. Interestingly, for all the plant parts studied, the infused extracts were the most effective metal chelator (Table 2). From the above findings, we found a considerable activity of the leaf extracts for the assays employed, which is in conformity with their levels of total alkaloids, TPC, TFC, and TPAC.

### 3.3. Enzyme Inhibitory Activity

In the present study, the cholinesterases (AChE and BChE), tyrosinase, α-amylase, and α-glucosidase were selected to determine the enzyme inhibitory potentials of the *C. szovitsii* subsp. *szovitsii* extracts and the results are illustrated in Table 3. Cholinesterase inhibitors are known to enhance cognitive function by inhibiting the enzymes that degrade acetylcholine in the brain and this approach is known as cholinergic hypothesis. Cholinesterase inhibitors have always been a significant therapeutic target for the treatment of Alzheimer’s disease [33]. As shown in Table 3, the methanolic extracts obtained using maceration and Soxhlet of the *C. szovitsii* subsp. *szovitsii* flowers, roots, and leaves were the most effective AChE inhibitors. No activity against AChE was observed for flower extracts obtained using the infusion and Soxhlet techniques. Of all the extracts studied, only the methanolic extracts obtained using the maceration and Soxhlet methods inhibited BChE with values of 6.72 and 3.11 mg GALAE/g, respectively.

Approximately 15% of the world population invest in skin whitening agents, with Asia dominating the market [34]. According to a survey by global industry analysts [35], the global market for skin lighteners will amount to $23 billion by 2020, driven by new markets particularly in India, Japan, and China. The molecular mechanism of these skin lightening agents is to decrease the melanin production that determines skin color. Tyrosinase is a key enzyme involved in melanin biosynthesis and therefore, inhibitors of this enzyme may lead to novel skin whitening agents that are safer and effective in comparison to the currently available ones [36]. From this standpoint, we assessed the anti-tyrosinase effect of the studied extracts and we observed that only the methanolic extracts obtained using the maceration and Soxhlet methods of *C. szovitsii* subsp. *szovitsii* flowers, roots, and leaves showed remarkable inhibition capacity against the tested enzyme.

New α-amylase and α-glucosidase inhibitors are also being sought to regulate post-prandial glucose levels because of the adverse side-effects linked to commercially available anti-diabetic medications. The findings (Table 3) revealed that all studied extracts displayed modest activity against α-amylase, with values ranging from 0.13 to 0.61, 0.11 to 0.49, and 0.10 to 0.61 mmol ACAE/g for the flowers, roots, and leaves, respectively. With respect to α-glucosidase, the methanolic extracts obtained using the maceration and Soxhlet methods of all parts of *C. szovitsii* subsp. *szovitsii*, except the macerated roots extract, were very potent.

### 3.4. Correlations

Pearson’s correlation coefficients were then calculated in order to check the contribution of polyphenols and alkaloids to the biological properties observed when considering the different *C. szovitsii* extracts. A summarizing table reporting all of the correlation coefficients for each part analyzed (i.e., flowers, roots, and leaves) is reported in the Supplementary Materials. Overall, the total alkaloids characterizing *C. szovitsii* roots were found to be highly (*p* < 0.01) correlated to ABTS (0.97), AChE inhibition (0.95), tyrosinase inhibition (0.96), and amylase inhibition (0.95) values. These findings were also supported by several researchers, who reported that alkaloids were the main players against cholinesterase [37,38] and tyrosinase [39,40]. Looking to polyphenols characterizing the root extracts, the most significant correlations (*p* < 0.01) were observed between anthocyanins and CUPRAC values (0.86), flavanols and tyrosinase inhibition (−0.99), flavones and CUPRAC values (0.85), phenolic acids and FRAP values (−0.87), tyrosols and tyrosinase inhibition (0.97), and stilbenes and DPPH values (0.93). Regarding *C. szovitsii* leaf extracts, we found no significant correlations between the total alkaloid content and the biological activities inspected (Supplementary Materials). However, polyphenols showed strong correlation coefficients, mainly when considering the in vitro antioxidant values. In this regard, excluding tyrosols, each polyphenol class analyzed was significantly correlated (*p* < 0.01) to the DPPH, FRAP, CUPRAC, and ABTS assays. In accordance with our results, polyphenols were reported as the main contributors to antioxidant properties in earlier studies [41,42]. On the other hand, only flavonoids (i.e., anthocyanins, flavanols, flavonols, and flavones) were found to be highly correlated to the enzymatic inhibition tested, with the most significant values (*p* < 0.01) recorded for flavanols (Supplementary Materials). These results are consistent with recent papers, which reported that flavonoids are one of the most important classes of natural enzyme inhibitors [43,44,45]. Finally, when considering the *C. szovitsii* flower extracts, we found negative correlation coefficients between the total alkaloids and the in vitro antioxidant assays (mainly with FRAP and ABTS values of −0.92 and −0.90, respectively). Regarding polyphenols characterizing flower extracts, significant correlations (*p* < 0.01) were obtained between anthocyanins and enzymatic inhibition assays, with the highest coefficient recorded for amylase inhibition (i.e., 0.99). These findings could be valuable for designing functional food ingredients and anthocyanins have exhibited promising antidiabetic effects in several studies [46,47]. In this case, flavanols were highly correlated to in vitro antioxidant properties, showing higher values for the CUPRAC and FRAP assays (on average: 0.99). This observation could be explained by the presence of the galloyl moiety at the C-3 position of flavonols, thus they exhibited a significant electron-donation ability [48]. Interestingly, lignans were found to be strongly and negatively correlated with the enzymatic assays (Table S1).

Clearly, the present correlation values depended on the parts of *C. szovitsii*. This fact may be explained by the different exposure of abiotic and biotic stress factors for each plant part and thus these parts could produce different levels of secondary metabolites. These findings were also supported by scientific evidence [49,50]. To sum up, our findings suggest that each part of *C. szovitsii* could be of interest for food, pharmaceutical, and cosmetic areas.

## 4. Conclusions

When considering previous studies regarding the pharmacological attributes of the *Colchicum* genus, there have been very few studies including *C. szovitsii* subsp. *szovitsii.* This study reports the biochemical characterization of *Colchicum szovitsii* subsp. *szovitsii,* according to their phenolic and alkaloid compositions, together with antioxidant and enzyme inhibitory activities. Of the plant parts studied, the extracts from leaves exhibited the highest phenolic levels associated with potent antioxidant and key enzyme inhibition associated with chronic pathologies, namely neurodegenerative complications (acetyl and butyrylcholinesterase), hyperpigmentation (tyrosinase), and diabetes mellitus (α-amylase and α-glucosidase). Detailed chemical profiling using UHPLC-QTOF mass spectrometry allowed us to confirm the wide phenolic and alkaloid distribution in the different extracts tested.

This work reinforces the notion that (poly)-phenols and alkaloids largely contribute to antioxidant and other pharmacological attributes, in the case of enzyme inhibition, making the studied species a promising source of drugs and whitening agents for medicine and cosmetics applications. In addition to fill the scientific gap on *C. szovitsii*, the presented findings advocate this species as a potential source for obtaining natural bioactive products for medical and pharmaceutical applications. However, further studies (toxicity, bioavailability, etc.) on *C. szovitsii* should be developed, aiming at replacing synthetic antioxidants and enzyme inhibitors.

## Figures and Tables

**Table 1:** Semi-quantitative values for the main phenolic sub-classes and total alkaloids by ultra-high-performance liquid chromatography quadrupole time-of-flight (UHPLC-QTOF) mass spectrometry of the tested extracts *.

Parts	Methods	Total Alkaloids	Anthocyanins	Flavanols	Flavones	Flavonols	Phenolic acids	Lignans	Tyrosols	Stilbenes
Flowers	Infusion	0.79 ± 0.03 ^a,b^	0.42 ± 0.01 ^c^	0.05 ± 0.00 ^d^	0.73 ± 0.01 ^b,c^	6.10 ± 0.03 ^c^	2.28 ± 0.17 ^b^	0.80 ± 0.03 ^c^	1.35 ± 0.50 ^b,c^	0.04 ± 0.01 ^b,c^
	Maceration-MeOH	0.84 ± 0.01 ^b^	0.60 ± 0.00 ^h^	0.09 ± 0.00 ^g^	1.19 ± 0.01 ^e^	9.89 ± 0.06 ^d,e^	3.27 ± 0.07 ^e,f^	0.04 ± 0.02 ^a^	1.08 ± 0.30 ^b^	0.01 ± 0.00 ^a^
	Maceration-Water	0.68 ± 0.05 ^a^	0.50 ± 0.05 ^e^	0.49 ± 0.00 ^h^	0.64 ± 0.01 ^b^	10.82 ± 1.30 ^e^	3.08 ± 0.04 ^d,e^	0.72 ± 0.11 ^b,c^	1.23 ± 0.02 ^b,c^	0.05 ± 0.00 ^c,d^
	Soxhlet-MeOH	0.85 ± 0.00 ^b^	0.58 ± 0.01 ^g^	0.08 ± 0.00 ^f^	0.81 ± 0.02 ^b,c,d^	9.25 ± 0.07 ^d^	2.90 ± 0.07 ^d^	0.02 ± 0.01 ^a^	0.69 ± 0.03 ^a^	0.03 ± 0.02 ^a,b^
Roots	Infusion	1.55 ± 0.02 ^d^	0.01 ± 0.00 ^a^	<0.01 ^a^	0.24 ± 0.00 ^a^	0.81 ± 0.00 ^a^	5.19 ± 0.06 ^i^	0.22 ± 0.12 ^a^	1.96 ± 0.05 ^d^	0.03 ± 0.00 ^a,b^
	Maceration-MeOH	1.82 ± 0.37 ^e^	0.01 ± 0.00 ^a^	nd	0.27 ± 0.01 ^a^	0.66 ± 0.02 ^a^	4.74 ± 0.05 ^h^	0.24 ± 0.05 ^a^	4.15 ± 0.03 ^g^	0.07 ± 0.01 ^d,e^
	Maceration-Water	1.39 ± 0.01 ^c^	0.01 ± 0.00 ^a^	<0.01 ^a^	0.15 ± 0.00 ^a^	2.10 ± 0.59 ^b^	2.55 ± 0.07 ^b,c^	0.18 ± 0.13 ^a^	1.05 ± 0.04 ^b^	0.06 ± 0.01 ^c,d^
	Soxhlet-MeOH	1.82 ± 0.01 ^e^	0.02 ± 0.00 ^a^	nd	0.81 ± 0.01 ^b,c,d^	1.58 ± 0.02 ^a,b^	4.05 ± 0.50 ^g^	0.31 ± 0.03 ^a^	3.94 ± 0.06 ^f g^	0.08 ± 0.01 ^e,f^
Leaves	Infusion	2.43 ± 0.25 ^f^	0.48±0.01 ^d^	0.04 ± 0.00 ^c^	1.14±0.01 ^d,e^	19.73 ± 0.21 ^f^	3.52 ± 0.13 ^f^	1.54 ± 0.02 ^d^	3.68 ± 0.08 ^e,f^	0.10 ± 0.00 ^f^
	Maceration-MeOH	2.45 ± 0.11 ^f^	0.58 ± 0.02 ^g^	0.06 ± 0.00 ^e^	1.03 ± 0.38 ^c,d,e^	21.65 ± 0.45 ^g^	2.83 ± 0.01 ^c,d^	0.39 ± 0.03 ^a,b^	3.41 ± 0.13 ^e^	0.09 ± 0.00 ^f^
	Maceration-Water	2.65 ± 0.02 ^g^	0.03 ± 0.01 ^b^	0.03 ± 0.00 ^b^	0.21 ± 0.01 ^a^	0.69 ± 0.09 ^a^	1.31 ± 0.03 ^a^	0.25 ± 0.03 ^a^	1.54 ± 0.09 ^c^	0.05 ± 0.00 ^c,d^
	Soxhlet-MeOH	2.46 ± 0.06 ^f^	0.56 ± 0.02 ^f^	0.06 ± 0.00 ^e^	1.71 ± 0.57 ^f^	21.92 ± 0.88 ^g^	2.56 ± 0.03 ^b,c^	1.07 ± 0.67 ^c^	3.65 ± 0.04 ^e,f^	0.10 ± 0.00 ^f^

* Values are reported as the mean ± standard deviation (*n* = 3). The results are expressed as mg equivalents/g dry matter. Different superscript letters in the same column indicate significant differences in the extracts (*p* < 0.05). nd = not detected.

**Table 2:** In vitro antioxidant activities of the tested extracts *.

Parts	Methods	Phosphomolybdenum (mmolTE/g)	DPPH (mgTE/g)	ABTS (mgTE/g)	CUPRAC (mgTE/g)	FRAP (mgTE/g)	Metal Chelating (mgEDTAE/g)
Flowers	Infusion	0.77 ± 0.02 ^e^	53.20 ± 0.33 ^g^	40.98 ± 0.38 ^g^	57.92 ± 1.37 ^e^	56.47 ± 0.27 ^g^	17.56 ± 0.52 ^g^
	Maceration-MeOH	0.67 ± 0.10 ^c,d^	43.47 ± 0.43 ^e^	29.39 ± 0.17 ^d^	64.19 ± 0.34 ^f^	53.71 ± 0.41 ^f^	7.72 ± 0.74 ^d^
	Maceration-Water	1.44 ± 0.03 ^f^	52.76 ± 1.54 ^g^	66.07 ± 0.90 ^i^	105.87 ± 0.63 ^h^	95.22 ± 0.58 ^h^	10.33 ± 0.95 ^e^
	Soxhlet-MeOH	0.62 ± 0.07 ^c,d^	47.73 ± 0.96 ^f^	33.56 ± 0.73 ^e^	62.20 ± 0.59 ^f^	55.50 ± 0.26 ^g^	5.53 ± 0.17 ^c,d^
Roots	Infusion	0.34 ± 0.02 ^a^	25.92 ± 0.06 ^a^	17.64 ± 0.62 ^b^	23.62 ± 0.41 ^a^	21.24 ± 0.16 ^a^	28.27 ± 0.74 ^i^
	Maceration-MeOH	0.50 ± 0.07 ^b^	30.57 ± 0.22 ^b^	24.71 ± 0.86 ^c^	31.92 ± 0.81 ^b^	27.15 ± 0.17 ^b^	6.01 ± 0.10 ^c,d^
	Maceration-Water	0.62 ± 0.01 ^c,d^	29.71 ± 0.16 ^b^	13.03 ± 0.64 ^a^	29.28 ± 0.12 ^b^	33.76 ± 1.00 ^d^	2.93 ± 0.30 ^a,b^
	Soxhlet-MeOH	0.69 ± 0.02 ^d,e^	33.04 ± 0.60 ^c^	28.28 ± 0.78 ^d^	40.23 ± 0.84 ^c^	31.58 ± 0.34 ^c^	4.94 ± 0.24 ^b,c^
Leaves	Infusion	0.63 ± 0.01 ^c,d^	84.59 ± 0.59 ^h^	66.63 ± 0.69 ^i^	92.29 ± 3.15 ^g^	96.00 ± 2.38 ^h^	35.45 ± 4.25 ^j^
	Maceration-MeOH	0.67 ± 0.06 ^c,d^	91.59 ± 0.33^j^	57.93 ± 1.77 ^h^	132.14 ± 5.51 ^i^	108.23 ± 1.69 ^i^	21.07 ± 0.31 ^h^
	Maceration-Water	0.62 ± 0.04 ^c,d^	36.52 ± 0.64 ^d^	38.55 ± 1.23 ^f^	46.30 ± 0.17 ^d^	43.50 ± 0.51 ^e^	2.15 ± 0.11 ^a^
	Soxhlet-MeOH	0.58 ± 0.02 ^c^	90.37 ± 0.21 ^i^	58.81 ± 1.06 ^h^	131.03 ± 0.59 ^i^	110.82 ± 0.16 ^i^	12.70 ± 0.34 ^f^

* Values are reported as the mean ± S.D. TE: Trolox equivalent; EDTAE: EDTA equivalent. Different superscript letters in the same column indicate significant differences in the extracts (*p* < 0.05).

**Table 3:** Enzyme inhibitory effects of the tested extracts *.

Parts	Methods	AChE Inhibition (mg GALAE/g)	BChE Inhibition (mgGALAE/g)	Tyrosinase Inhibition (mg KAE/g)	Amylase Inhibition (mmol ACAE/g)	Glucosidase Inhibition (mmol ACAE/g)
Flowers	Infusion	na ^a^	na ^a^	na ^a^	0.13 ± 0.01 ^b^	na ^a^
	Maceration-MeOH	4.22 ± 0.30 ^g^	na ^a^	99.94 ± 1.20 ^b^	0.61 ± 0.01 ^f^	2.65 ± 0.08 ^c^
	Maceration-Water	na ^a^	na ^a^	2.46 ± 0.19 ^a^	0.33 ± 0.02 ^d^	na ^a^
	Soxhlet-MeOH	3.82 ± 0.17 ^f^	na ^a^	103.19 ± 2.78 ^c^	0.50 ± 0.01 ^e^	2.65 ± 0.06 ^c^
Roots	Infusion	0.23 ± 0.06 ^b^	na ^a^	na ^a^	0.11 ± 0.01 ^a,b^	na ^a^
	Maceration-MeOH	4.21 ± 0.22 ^g^	6.72 ± 0.23 ^c^	113.00 ± 1.24 ^d,e^	0.49 ± 0.01 ^e^	2.79 ± 0.01 ^d^
	Maceration-Water	0.60 ± 0.04 ^c^	na ^a^	na ^a^	0.13 ± 0.01 ^b^	na ^a^
	Soxhlet-MeOH	4.48 ± 0.05 ^h^	3.11 ± 0.04 ^b^	112.63 ± 4.31 ^d^	0.49 ± 0.01 ^e^	na
Leaves	Infusion	0.29 ± 0.06 ^b^	na ^a^	na ^a^	0.17 ± 0.01 ^c^	na ^a^
	Maceration-MeOH	3.43 ± 0.08 ^e^	na ^a^	116.92 ± 1.28 ^f^	0.59 ± 0.04 ^f^	2.49 ± 0.10 ^b^
	Maceration-Water	0.93 ± 0.03 ^d^	na ^a^	na ^a^	0.10 ± 0.01 ^a^	na ^a^
	Soxhlet-MeOH	3.88 ± 0.10 ^f^	na ^a^	115.61 ± 1.03 ^e,f^	0.61 ± 0.03 ^f^	2.54 ± 0.01 ^b^

* Values are reported as the mean ± S.D. Different superscript letters in the same column indicate significant differences in the extracts (*p* < 0.05). GALAE: Galatamine equivalent; KAE: Kojic acid equivalent; ACAE: Acarbose equivalent; na: not active.

## Supplementary Materials

The following are available online at https://www.mdpi.com/2076-3921/8/12/632/s1, Table S1: dataset containing all polyphenols and alkaloids putatively annotated together with Pearson’s correlation coefficients, VIP marker compounds from OPLS-DA models, and in vitro spectrophotometric assay results (i.e., TPC, TFC, TPA).
